# The contributions of rainfall and fog to leaf water of tree and epiphyte communities in a tropical cloud forest

**DOI:** 10.3389/fpls.2024.1488163

**Published:** 2024-10-17

**Authors:** Qingqing Yang, Zijing Zhang, Hui Zhang, Huai Yang, Shree Pandey, Robert John

**Affiliations:** ^1^ School of Ecology, Hainan University, Haikou, China; ^2^ Hainan Academy of Forestry (Hainan Academy of Mangrove), Haikou, China; ^3^ Key Laboratory of Tropical Forestry Resources Monitoring and Application of Hainan Province, Haikou, China; ^4^ Key Laboratory of Genetics and Germplasm Innovation of Tropical Special Rainforest Trees and Ornamental Plants (Hainan University), Ministry of Education, School of Tropical Agriculture and Forestry, Hainan University, Haikou, China; ^5^ Hainan Institute of National Park, Haikou, China; ^6^ Institute of Tropical Bamboo, Rattan & Flower, Sanya Research Base, International Center for Bamboo and Rattan, Sanya, China

**Keywords:** hydraulic response, leaf water supply, isotope, photosynthesis rate, transpiration rate

## Abstract

**Introduction:**

Tropical cloud forest ecosystems are expected to face reduced water inputs due to climatic changes.

**Methods:**

Here, we study the ecophysiological responses of trees and epiphytes within in an Asian cloud forest to investigate the contributions of rainfall, fog, and soil to leaf water in 60 tree and 30 vascular epiphyte species. We measured multiple functional traits, and δ^2^H, and δ^18^O isotope ratios for leaf water, soil water, rainfall, and fog in the wettest (July) and driest (February) months. Using a Bayesian stable isotope mixing model, we quantified the relative contributions of soil water, fog, and rainfall to leaf water.

**Results and discussion:**

Rainfall contributes almost all the leaf water of the epiphytes in July, whereas fog is the major source in February. Epiphytes cannot tap xylem water from host trees, and hence depended on fog water when rainfall was low. Most of leaf water was absorbed from soil water in July, while fog was an important source for leaf water in February despite the soil moisture content value was high. In February, lower temperatures, along with reduced photosynthesis and transpiration rates, likely contributed to decreased soil water uptake, while maintaining higher soil moisture levels despite the limited rainfall. These contrasting contributions of different water sources to leaf water under low and high rainfall and for different plant groups outline the community-level ecophysiological responses to changes in rainfall. While direct measurements of water flux, particularly in roots and stems, are needed, our results provide valuable insights on tropical cloud forest hydrology under scenarios of decreased fog immersion due to climatic changes.

## 1 Introduction

Tropical cloud forests, despite occupying only 1.4% of the world’s tropical forest area (Scatena et al., 2011), there is disproportionately high diversity and endemism for plant and animal species (Bruijnzeel et al., 2010; Karger et al., 2021). They occur in mountains where cloud or fog immersion of the forest canopy is a frequent phenomenon, and where plants benefit from the foliar uptake of ‘occult’ precipitation (i.e., mist, cloud water, fog, fine drizzle and wind-driven rain), at least during the dry season (Bruijnzeel et al., 2010). Due to their distinct climatic and hydrological, tropical cloud forests are widely regarded as sensitive to climatic change response. This was evident in the dramatic declines and extinctions of amphibian species in the cloud forests of Central America in the 1990s due to warming-induced drought stress, which drew global attention to the vulnerability of these ecosystems to biodiversity loss (Pounds et al., 1999; Alan Pounds et al., 2006; Fisher and Garner, 2020). Model projections (Still et al., 1999; Helmer et al., 2019) and empirical evidence (Ray et al., 2006; Nair et al., 2008; Diaz et al., 2011; Krishnaswamy et al., 2014; Los et al., 2021) support the hypothesis of ‘lifting cloud base’ and drying in tropical montane environments in response to climate warming, with projected negative impacts on hydrology due to reduced cloud immersion (Hildebrandt and Eltahir, 2007; Hu and Riveros-Iregui, 2016; Los et al., 2021). Despite the existing knowledge, the variability observed in cloud forest types and the documented ecohydrological patterns (Hu and Riveros-Iregui, 2016) highlight the necessity for further studies encompassing diverse ecosystems and major taxonomic groups, particularly in cloud forest sites with seasonal rainfall patterns and potential drought stress.

Tropical cloud forests exhibit a remarkable diversity and structural complexity in their vegetation and ecohydrological attributes, yet research efforts have not been evenly distributed across all cloud forests. Climatic stressors are already changing the ecohydrological conditions of cloud forests, with negative impacts on regional watersheds and fresh-water supply, particularly in sites that face seasonal water stress (Hildebrandt and Eltahir, 2007; Los et al., 2019). Rising temperatures in tropical montane regions can alter the spatial-temporal dynamics of fog occurrence and reduce cloud water interception by vegetation, resulting in loss of moisture inputs (Hu and Riveros-Iregui, 2016; Los et al., 2019; Li et al., 2022). Combined with other factors such as strong climatic variation and shallow soils, the impact of reduced cloud water interception on tree populations (Werner, 1988; Aiba and Kitayama, 2002; Anchukaitis and Evans, 2010) will depend on plant hydraulic responses to water availability (Anderegg et al., 2012; Berry et al., 2015; Choat et al., 2018). Besides, the importance of ‘occult’ precipitation in alleviating any decline in soil water availability due to decreasing rainfall or seasonal drought stress needs to be quantified across cloud forest sites.

The first step in understanding the importance of ‘occult’ precipitation in tropical cloud forests is to examine fog-induced specific hydraulic responses of plant species. Previous studies have only been explored within subsets of species (Burgess and Dawson, 2004; Johnson and Smith, 2008; Ritter et al., 2009; Eller et al., 2013, 2016; Gotsch et al., 2014, 2018; Binks et al., 2019; Cavallaro et al., 2020), thereby constraining our comprehension of community-level responses. As a phenomenon, foliar uptake of fog water has been reported widely, but the relative contribution of fog uptake to leaf water may vary considerably within and among individuals and species (Burgess and Dawson, 2004; Limm et al., 2009; Goldsmith et al., 2013). Foliar fog water uptake may also depend on rainfall distribution, fog duration, and plant life-form (e.g., trees vs. epiphytes) (Oliveira et al., 2014; Gotsch et al., 2014; 2015; Wu et al., 2018; Berry et al., 2015, 2019; Binks et al., 2019). An effective assessment of community-level hydraulic responses to changes in fog incidence would require a wide coverage of the species and life forms in cloud forests (Suding et al., 2008; Laughlin, 2014).

In this study, we quantify the contributions of rain, fog, and soil waters to vegetation in an old-growth tropical cloud forest in Hainan Island, southern China. The tropical cloud forests in Hainan Island are stunted (‘elfin’ forests) and experience highly seasonal rainfall (Long et al., 2011b). The basic fog induced plant hydraulic responses are unknown and we cannot yet assess the impact of climatic changes on cloud forest vegetation in this region. We argue that quantifying community-level responses (such as possible differences in photosynthesis, transpiration, and soil- and foliar/fog water uptake) between seasons (dry winter vs. wet summer) and life-forms (e.g., trees vs. epiphytes) are the key to understanding how this tropical cloud forest plant community copes with seasonal or temporal changes in water availability. Therefore, we evaluated community-wide ecophysiological responses of the trees and epiphytes to changes in water availability by examining the following: (i) leaf isotope ratios (δ^2^H, δ^18^O and δ^13^C) and several key functional traits (transpiration rate, leaf turgor loss point, leaf hydraulic capacitance and photosynthesis rate) for 60 tree species and 30 vascular epiphyte species in the wettest and driest months; (ii) the isotope ratios (δ^2^H and δ^18^O) in soil water, rain, and fog water in the wettest and driest months; (iii) species abundances of 60 tree species and 30 vascular epiphyte species in 21 plots of 400 m^2^ each, and (iv) soil water content in the peak months of the wet and dry seasons.

Our study design, and the selection of ecophysiological measurements are based on hypothesized plant responses to seasonal changes in precipitation. Fog, along with fine drizzle (wind-driven rain), increases water inputs to the ecosystem (Bruijnzeel et al., 2011; Giambelluca and Gerold, 2011; Binks et al., 2019). However, it may also exert an influence on water movement within plants by diminishing solar radiation and temperature, which subsequently impedes photosynthesis, transpiration, and soil water absorption by roots (Goldsmith et al., 2013; Weathers et al., 2020). This might explain in part why cloud forests maintain high soil water content (Goldsmith et al., 2013; Muñoz-Villers and McDonnell, 2013; Dawson and Goldsmith, 2018; Gerlein-Safdi et al., 2018), even during the dry season (Bruijnzeel and Veneklaas, 1998). Nevertheless, a predicted 2°C warming may elevate the cloud-base heights by 250 m (predictions for 2052 by IPCC 5th assessment reports) with differential impact on plant growth forms (trees, grasses, epiphytes) (Nadkarni and Solano, 2002; Zotz and Bader et al., 2009; Wu et al., 2018; Liu et al., 2005), and result in a significant loss of tropical cloud forest cover in some sites (Los et al., 2019; de Meyer et al., 2022).

We tracked hydrological inputs through leaf isotope ratios (δ^2^H and δ^18^O), which when analyzed with a Bayesian stable isotope mixing model, can trace the relative contribution of rainfall, soil, and fog to leaf water in both trees and epiphytes (Wu et al., 2018; Wang et al., 2019). We then used several ecophysiological traits including leaf-level photosynthesis and transpiration rates, leaf carbon isotopic composition (δ^13^C) as an indicator of long-term intrinsic water use efficiency (Cernusak et al., 2013; Ellsworth and Cousins, 2016; Acosta-Rangel et al., 2018), leaf turgor loss point (TLP) (Bartlett et al., 2012; Werner, 1988; Jarvis and Mulligan, 2011), and leaf hydraulic capacitance (Brodribb and Holbrook, 2003), all of which capture critical plant hydraulic responses. We therefore investigated (1) whether there is differential contribution to leaf water between species, life forms, and seasons from the three possible sources of leaf water - soil, rainfall, and fog; (2) whether community-level ecophysiological responses vary between the wettest and driest months and between life forms; and (3) how these ecophysiological responses help to maintain the hydraulic safety of vegetation and the ecohydrology of this cloud forest plant community.

## 2 Materials and methods

### 2.1 Study site

The study was conducted in a tropical montane evergreen dwarf cloud forest (‘elfin’ forest; tropical cloud forest) at Bawangling Area of Hainan Tropical Rainforest National Park (109°05′-109°25′E, 18°50′-19°05′N), located in Hainan Island, southern China (Long et al., 2011b). This region belongs to the tropical monsoon climate area with a mean annual rainfall of ~2500 mm, and a distinct wet season from May to October, which accounts for about 80% of the annual rainfall. In 2017, a local meteorological station was established near our experimental site and observations during the 2018 show that the mean monthly rainfall during the wet season was 306 mm, with July emerging as the wettest month with a rainfall of 375 mm (**Supplementary Figure S1**). The lowest rainfall (58 mm) and frequency (5 days) of rain were observed for February. The dry season (monthly rainfall<100 mm), spanning a period of five months from November to March, is characterized by monthly average rainfall below 78 mm (see also Long et al., 2011b). April was relatively wetter with 138 mm of rain, after which the wet season begins in May. Based on these meteorological data, effective monthly rainfall (monthly rainfall – monthly potential evapotranspiration) was +235.7 mm for July and -16.7 mm for February, indicating only a slight rainfall deficit in the driest month.

The reserve is predominantly comprised of old-growth tropical cloud forest, on a substrate of lateritic soil developed primarily from sandstone bedrocks (Cheng et al., 2020). These forests typically occur as mountaintop islands over 1250 m above sea level, where terrain slope range from 3° to 65° (Long et al., 2011a). The sites typically have very shallow (30-70 cm) soil, high spatial extent (40%) of exposed rock, and very short tree root length (less than 30 cm) (Long et al., 2011c; Yang et al., 2021). The dominant plant species include *Distylium racemosum, Syzygium buxifolium, Xanthophyllum hainanense, Camellia sinensis* var. *assamica and Cyclobalanopsis championii*. The average tree height in these forests is rather low at 4.8 ± 2.8 m, but as is typical of cloud forests, average tree density is high at 9633 stems ha^-1^ (Long et al., 2011b). A total of 89 tree species have been recorded in 41 plots of 100 m^2^ each (Long et al., 2011a).

### 2.2 Field sampling

We carried out field sampling and measurements in the months of February and July of 2019, primarily to quantify the contributions of various water sources to leaf water in tree and epiphyte species in the wettest and driest months of the year (**Supplementary Figure S1**). The sampling was carried out in 21 vegetation plots, each of size 20 × 20 m^2^, which we had established in previous work (Long et al., 2011b). These plots are located within a narrow elevation range of 1313 m to 1395 m above mean sea level (Long et al., 2011b; **Supplementary Figure S2** and **Supplementary Table S1**). The plots are separated from each other by about 100 m and do not show significant spatial autocorrelation in species abundances or soil properties (Long et al., 2015). The total area of the tropical cloud forest in the reserve is just about 3 hectares, and the 21 plots are scattered as widely as possible across this mountaintop patch (**Supplementary Figure S2**).

In this study, we recorded all freestanding trees with a diameter of ≥ 1 cm at breast height (DBH) within each plot and identified them to species. The relatively low tree height in this cloud forest allowed us to accurately measure species abundance (total number of individuals) for all epiphyte species on the host trees in the 21 plots. We followed the method proposed by Sanford (1968) to record the species abundances for all vascular epiphytes in the plots. The specific details are given in **Supplementary Data Sheet - Text S1** in the **Supplementary Material**.

To measure the contribution of different water sources to leaf water in the wettest and driest month of the year, we measured hydrogen and oxygen isotope ratios (δ^2^H and δ^18^O) in leaf water (for tree and epiphyte communities), rain water, soil water, and fog drip water, for both of these seasons. We also measured soil water content using standard gravimetric analysis. For isotope measurements of leaf water, we sampled 20 mature, healthy, sun-exposed canopy leaves from three to five individuals of each tree and epiphyte species present in the 21 plots. Due to the evaporative enrichment of the heavier isotope in leaves, stem xylem water isotope ratios may provide a better integrated signal of plant water (Zhao et al., 2016). However, given the challenges associated with sampling stem water, particularly for epiphytes, we focus our study on leaf water. Many epiphyte species in our tropical cloud forest are orchids with pseudobulb stems, so extracting their stem xylem water would have been difficult (Wang et al., 2019). We note that evaporative enrichment is lower in cloud forests because leaf wetting, fog, and high atmospheric humidity reduce transpiration rates (Alvarado-Barrientos et al., 2014). Finally, leaf water analysis may be a relatively non-intrusive method for quantifying the sources of plant water (Benettin et al., 2021).

We also collected water samples of fog drip and rainfall within each plot purely to measure isotope ratios (and not to quantify fog precipitation). We followed Liu et al. (2005) for fog drip collection, wherein a simple self-made fog drip collector made of plastic film was used to intercept and collect the fog droplets formed upon contact (**Supplementary Figure S3C**). Specifically, fog drip in each plot was collected by hanging a clean plastic film between the two trees, with clear exposure on the windward side (**Supplementary Figures S3B, C**). We set this from 19:00 to 21:00 h when heavy fog had set in and when there was no rain. The intercepted smaller fog droplets condense and gradually coalesce to form large water droplets, which are collected in a storage tank at the lower end of the plastic film. Finally, each condensed water sample was saved in a 20 ml tube. We collected fog drip water, soil water, and rain water separately. Rainfall samples were collected under the trees using a 20 ml tube at the beginning of a rainfall event. All the collected plant, soil, fog, and rainfall samples were stored in liquid nitrogen containers before measuring isotope ratios (δ^2^H, and δ^18^O). Due to the shallow soils and short tree root length, we sampled soils at 0-20 cm depth, collecting three samples (20 g each) in each plot, using a 5 cm diameter soil auger, in both the months. We collected soil water on a day when there was no rain. Before performing the isotope analysis, we needed to extract leaf water and soil water from all the samples, which we did using a fully automatic vacuum condensation extraction system (LI-2100, LICA United Technology Limited, Beijing, China). The extraction rate of water from the samples was assessed to be more than 98% on the basis of gravimetric analysis.

For plant trait measurements, undertaken in February and July, we severed a small branch from each tree being sampled and measured a set of plant traits including maximum photosynthesis rate (*A_area_*; μmol cm^-2^ s^-1^), transpiration rate (mol m^-2^ s^-1^), stomatal density (number of stomata mm^-2^ of leaf area), stomatal conductance (mmol m^-2^ s^-1^), leaf turgor loss point (TLP; MPa), and leaf δ^13^C for tree and vascular epiphyte communities. To minimize intraspecific variation in trait measurements, we selected only individuals with DBH near the species mean value. We also ensured that leaves were collected from the same individuals for each species in both the wettest and driest months.

For the dry season measurements, we carried out the sampling and field measurements from February 5 to 10, 2019, during which period rainfall occurred only on February 10. We matched these measurements for the wettest month and started sampling on 5^th^ July and completed the field work by the 10^th^ of July. Although it rained almost every day in the month of July, we had only night rains from July 5 to 6, 2019. Thus, we collected soil water samples and leaf samples (for measuring leaf isotope) in the mornings of July 5 and 6 for the wet season. We collected fog drip from 19:00 to 21:00 h on the nights of July 8 and February 8 and rainfall samples on July 10 and February 10. This study design enabled us to quantify 1) the hydraulic responses of the tree and epiphyte communities in the wettest and driest parts of the year; and 2) the relative contributions of rainfall, fog, and soil water to the water use strategies of tree and epiphyte communities in wettest and driest conditions.

### 2.3 Isotope (δ^2^H, δ^18^O, and δ^13^C) measurements

We measured δ^13^C using the conventional Pee Dee Belemnite standard (Farquhar et al., 1989). Then, we sampled 0.5-1.5 ml of leaf water, soil water, fog and rainfall, to measure δ^18^O and δ^2^H. The isotopic compositions were analyzed using a liquid water isotope analyzer (Model DLT-100, Los Gatos Research, USA) that employs cavity enhanced absorption spectroscopy. The precision of the isotope analyzer was typically better than ± 1.2‰ for δ^2^H and ± 0.3‰ for δ^18^O. To account for the possible presence of organic contaminants in cryogenically extracted water samples from plant tissues, the stable isotopic ratios of leaf water measured by the LGR system were corrected as described in previous studies (Schultz et al., 2011; Wu et al., 2016), and the specific details are given in **Supplementary Data Sheet - Text S2** in the **Supplementary Material**.

### 2.4 Meteorological variables and soil water measurements

In 2017, an automatic weather station (YT-QXC4, Shandong, China) was installed in a central location, near the 21 sampling plots (also in forested area) and situated at an elevation of 1245 m a.s.l. (**Supplementary Figure S2**). This installation enabled the continuous monitoring and collection of data - rainfall frequency (number of rainy days in a month), and monthly mean values for rainfall, total solar radiation, air temperature, humidity and wind speed. We also surveyed fog duration or timing from 6:00 to 23:00 h in July and February by observing for fog on each day. Specifically, nocturnal fog was observed by setting up a light in the plot. We used about 20 g soil for each of the 21 soil samples to measure gravimetric soil water (g/kg). All soil samples were oven-dried for 24 h at 105 °C for these measurements.

### 2.5 Maximum photosynthesis rate, transpiration rate and stomatal conductance, stomatal density, leaf hydraulic capacitance and leaf turgor loss point measurements

We used the Li-6800 portable photosynthesis system (Li-Cor, Lincoln, Nebraska, USA) to measure maximum photosynthesis rate, transpiration rate and stomatal conductance between 9:00 AM and 11:00 AM on sunny days. Five canopy leaf-bearing branches at similar heights (~20 mm in diameter) were harvested, and then photosynthetic measurements were taken within 1 h (Wyka et al., 2012). Based on preliminary trials, we set the photosynthetic photon flux at 1200 μmol m^-2^ s^-1^ to ensure all the species were measured for light-saturated photosynthetic rates (Zhang et al., 2018). We set chamber CO_2_ and air temperature as 400 μmol mol^-1^ and 28 °C, respectively. Before collecting the data, we first exposed the leaves to the above conditions for about 5 minutes to allow photosynthetic parameters to stabilize. We sampled five to six fully expanded and sun-exposed leaves from three to five mature individuals to measure maximum photosynthesis rate, transpiration rate and stomatal conductance, whose values are referred to as leaf area units.

Stomatal density was measured using the protocol in Carins Murphy et al. (2012). We first collect leaf surface film and then use an optical microscope (LEICA DM3000 LED) and Image J software to calculate stomatal density in the leaf cuticles (2 per leaf and 5 fields of view per cuticle).

Leaf turgor loss point was determined from leaf pressure-volume (P-V) curve (Sack et al., 2003) for each species in both seasons. For the measurement of P-V curve, we selected healthy leafy branches (or entire plants for several small epiphyte species) from five individuals in the early morning (05:00 and 07:00 h). The samples were packed in black plastic bags with the cut ends maintained underwater, and were immediately sent to the laboratory (within 1 h). The sampled leaves were water saturated because leaf water potential was higher than -0.3 MPa, and did not show a decrease during transportation. For each measured leaf, we determined saturated leaf mass and subsequently conducted a bench-drying procedure (dehydration on a lab bench at 25°C) to obtain a range of leaf water potentials. During leaf desiccation, we periodically measured leaf mass and leaf water potential (*K*_leaf_) by using a precision scale (0.0001g) and a pressure chamber (PMS, Corvallis, OR, USA), respectively. Finally, the dry leaf mass was determined after drying in an oven at 70°C for about 72 h. We calculated relative water content (RWC) and then constructed P-V curve by plotting leaf RWC against *K*_leaf_. Leaf P-V curves of the measured species showed distinct two-phase linear equation, and leaf water potential at turgor loss point (TLP) was estimated from the point of intersection of the two lines (Brodribb and Holbrook, 2003; Wang et al., 2021).

Leaf hydraulic capacitance (C) was determined from the slope of P-V curve for each species, with the help of following equation (Brodribb and Holbrook, 2003).


$$\text{C}=\text{δRWC}/{\text{δΨ}}_{\text{l}}\times \left(\text{DW}/\text{LA}\right)\times \left(\text{WW}/\text{DW}\right)/\text{M}$$

Here, DW is leaf dry weight (g), LA is leaf area (m^2^), WW is mass of leaf water at 100% RWC (g), and M is molar mass of water (g mol^-1^). δRWC/δΨ_l_ could be attained from P-V curve.

### 2.6 Statistical analysis

Community weighted mean (CWM) of a functional trait is defined as the summation over all species in the community of the species mean trait values weighted by their respective relative abundances (Garnier et al., 2004). CWM is considered a good measure of ecosystem response to abiotic factors (Lavorel and Garnier, 2002; Suding et al., 2008; Laughlin, 2014). Therefore, for each of the 21 plots, community-level trait metrics or isotopes (CWM*_jk_*) for the tree and epiphyte community were quantified for each trait or isotopes (*j*) in each plot (*k*) following Garnier et al. (2004) and Buzzard et al. (2016) and using the following formula:


$$CWM_{jk}=\sum x_{ik}t_{ik}$$

where *x_ik_* is the relative abundance of species *i* in plot *k* and *t_ik_* is the isotope or leaf functional trait value of species *i* in plot *k*. In reality, CWM*_jk_* was calculated using function ‘dbFD’ in the *FD* package in R.

The Bayesian stable isotope mixing model (Parnell et al., 2013) is widely used for tracing the proportional contributions of various sources to a stable isotope mixture. It is based on isotopic mass conservation and that the isotopic mass for the mixture should contain the signature of the proportional contributions of the isotopic masses from all its potential sources (Wang et al., 2019). As long as the multiple isotope sources have clearly different isotope ratio signatures, one can reliably apportion the contributions from these sources to a mixture. The Bayesian approach can incorporate priors such as rooting depth and other attributes that affect the relative contribution of different sources. In the cloud forest ecosystem, leaf water in trees can originate from soil water, fog, and rainfall, whereas the epiphytes we studied can access water only from the air via rainfall and fog and cannot tap into the host tree xylem water. Thus, by using Bayesian stable isotope mixing model and the isotope ratios (δ^2^H and δ^18^O) for leaf water, soil water, fog, and rainfall, we could trace the proportional contributions of soil water, rainfall, and fog to foliar/leaf water (Wu et al., 2018). This model was implemented using the ‘siarmcmcdirichletv4’ function in R (*siar* package), an algorithm which uses the distinctive δ^2^H and δ^18^O isotope ratios to quantify the relative contributions (%) of fog water, rainfall, and soil water to leaf water in tree and the epiphyte communities in the 21 plots for both the wettest and driest months.

We performed a series of comparisons to understand the differences in functional traits between life forms, differences in meteorological variables between seasons, and the impact of fog on plant physiology. We used a Wilcoxon Rank Sum test to compare differences in CWMs of maximum photosynthesis rate, transpiration rate and stomatal conductance and density for the tree and the epiphyte communities in the wettest and driest months. Further, we compared four meteorological variables (mean rainfall, total solar radiation, mean air temperature, and mean relative atmosphere humidity), and the soil water content between the two seasons. We also used Wilcoxon Rank Sum to compare the differences in CWMs of δ^13^C and leaf turgor loss point (TLP) between tree and epiphyte communities during the wettest and driest months. The objective of this analysis was to examine whether the tropical cloud forest ecosystems can maintaining a sufficient water supply in the driest month. Additionally, we determined whether there were any significant differences in CWMs of δ^13^C and leaf turgor loss point between the tree and epiphyte communities.

## 3 Results

### 3.1 Variations in four meteorological variables and soil water content in the wettest and driest months

**Figure 1:**
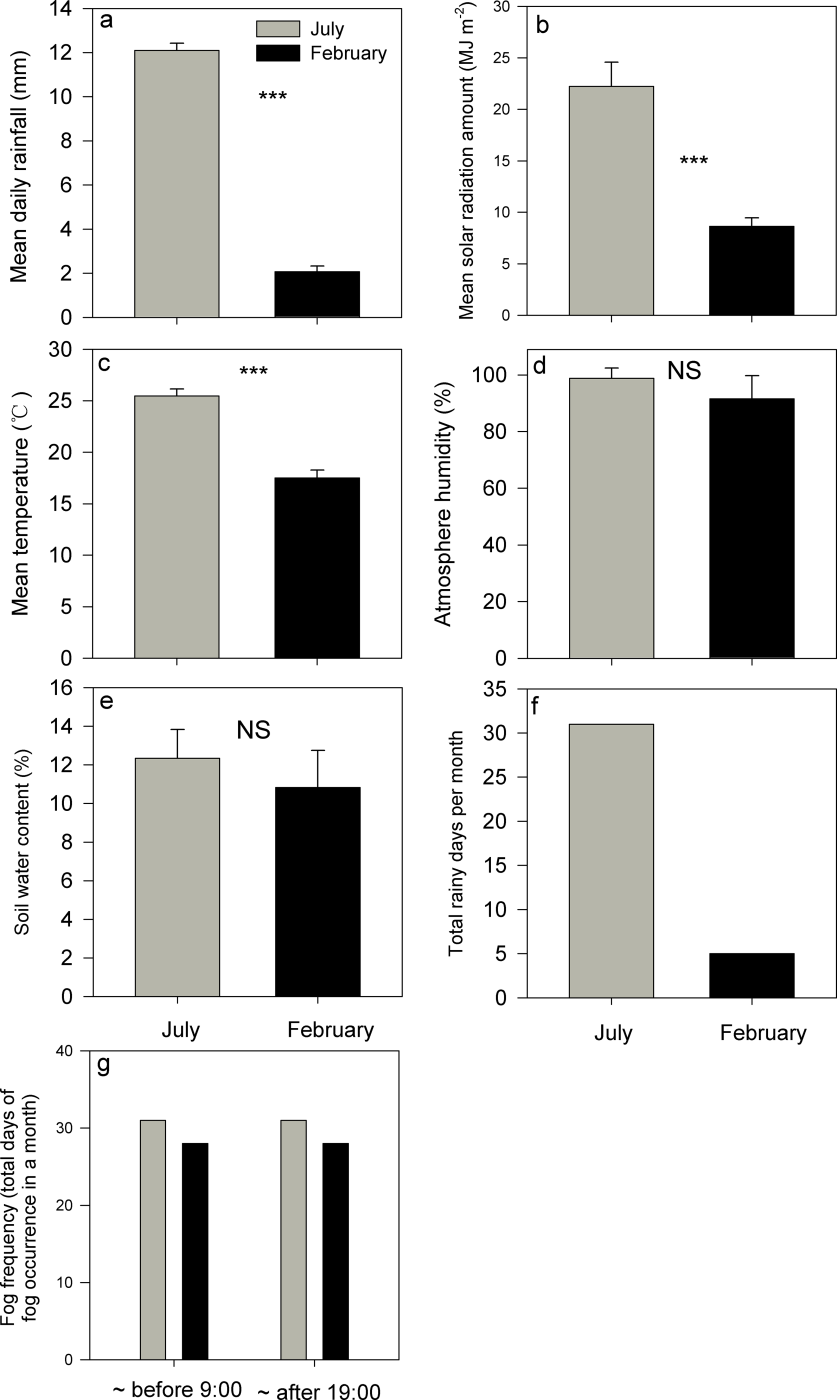
Evaluations of four below-canopy meteorological variables [**(A–D)**, mean daily rainfall, mean solar radiation amount, mean atmosphere temperature, and mean atmosphere humidity], **(E)**, soil water content, rainfall frequency [**(F)**, total rainfall days in a month] and fog frequency [**(G)**, the total days of fog occurrence in a month] in the July and February respectively. *** indicates significant differences at P<0.001, whereas NS indicates that the differences were not statistically significant (P>0.05) based on Wilcoxon signed-rank tests. Bars indicate the mean values; error bars denote standard errors.

In July, the mean daily rainfall, total solar radiation, and mean atmospheric temperature were recorded 12.11 mm, 22.23 MJ m^-2^, and 25.46°C. Conversely, in February, these values decrease to 2.07 mm, 8.64 MJ m^-2^, and 17.49°C (**Figures 1A–C**; **Supplementary Figure S1**). both July and February exhibited mean relative atmospheric humidity levels exceeding 90%, with July registering a slightly higher value of 98.63% compared to 91.53% in February. However, this discrepancy did not reach statistical significance (**Figure 1D**, *P*>0.05). Overall, soil water content was also not significantly different between July and February (**Figure 1E**, *P*>0.05). Rainfall occurred every day in July, whereas there were just five days of light rain in February (**Figure 1F**). No discernible differences in fog occurrence were observed between July and February, as fog was consistently present during early morning hours (before 9:00 h) and night-time (after 19:00 h) daily in both months (**Figure 1G**). In other words, the driest month is marked by much lower rainfall, attenuated total solar radiation, and lower temperature, while some key attributes like fog frequency, air humidity, and soil water content were not significantly lower in this month compared to the values in the wettest (summer) month (**Figure 1**).

### 3.2 Species compositions of the tree and the epiphyte communities

We studied a total of 60 tree and 30 vascular epiphyte species belonging to a total of 38 families across the 21 plots (**Supplementary Table S2**). The common tree species (relative abundance >5%) include *Distylium racemosum, Psychotria rubra, Syzygium buxifolium, Ervatamia officinalis*, and *Symplocos poilanei*, and the dominant vascular epiphyte species were *Eria thao, Coelogyne fimbriata, Liparis delicatula*, and *Bulbophyllum retusiusculum* (**Supplementary Table S2**). Our sampled epiphyte community could be considered as totally dependent on atmospheric water (rain, fog, dew) for their leaf water, as all the epiphyte species in the 21 plots were anchored on the tree stems but could not tap into the tree xylem water (**Supplementary Table S2**).

### 3.3 Variations of δ^2^H and δ^18^O compositions in soil water, rainfall, and fog

**Figure 2:**
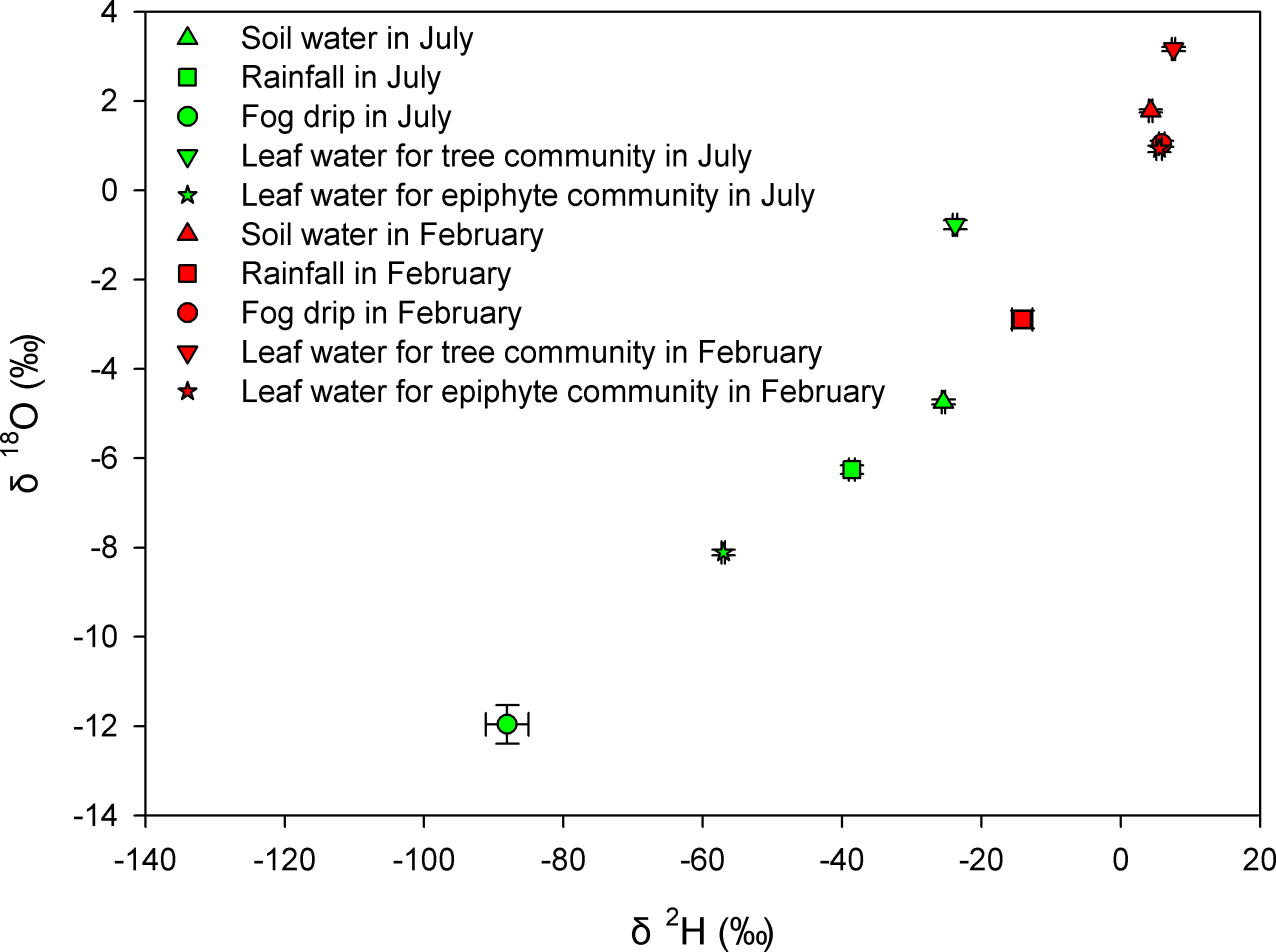
The dual isotope (δ^2^H and δ^18^O) plot for soil water, rainfall, fog drip, and leaf water for tree and epiphyte communities in July and February respectively.

As evident from the scatter plot in **Figure 2**, there were notable variations in the δ^2^H and δ^18^O isotopic values across various water sources (including soil water, rainfall, fog, and leaf water), between the tree and epiphyte communities, and between the months of July and February (**Figure 2**). Notably, the leaf water isotope values were different between seasons for both trees and epiphytes (**Figure 2** and p<0.001, Wilcoxon signed-rank tests, **Supplementary Table S3**). An important exception being δ^2^H and δ^18^O values of leaf water for the tree community, which were similar to the values for soil water in the wettest month (**Figure 2**, p>0.05, Wilcoxon signed-rank tests, **Supplementary Table S3**). Another exception was for leaf δ^2^H and δ^18^O values for the epiphyte community, which were similar to the values for rainfall in July and fog in February (**Figure 2**, p>0.05, Wilcoxon signed-rank tests, **Supplementary Table S3**).

### 3.4 The relative contributions of soil water, rainfall, and fog to foliar water resources

**Figure 3:**
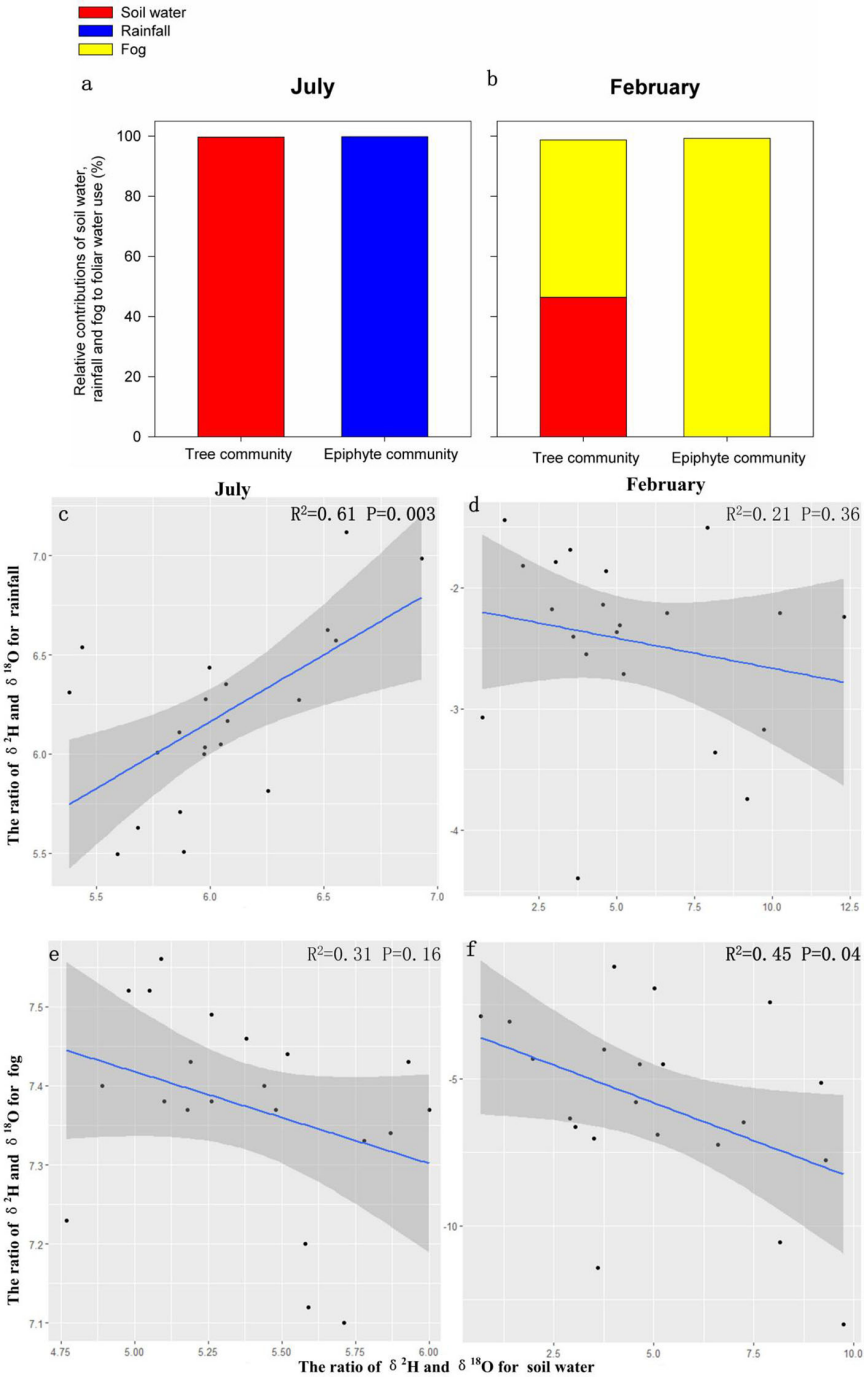
Relative contributions (%) of soil water, fog water, and rainfall towards foliar water resources for the tree and the epiphyte communities **(A, B)** and relationships among the ratio of δ^2^H and δ^18^O for soil water content, fog water and rainfall **(C)**. Contributions and correlations were assessed in July and February respectively **(D-F)**.

Given the high and persistent rainfall in the wettest (summer) month, we found that soil water contributed nearly 100% of the leaf water for tree communities and rainfall contributed nearly all the leaf water for epiphyte communities (**Figure 3A**). The contribution of fog water to foliar water uptake was insignificant for the tree or the epiphyte community in the wettest summer month (**Figures 3A, B**). The contribution of soil water to leaf water declined to 46.4% for the tree community despite no decrease in soil water content in the dry/winter month. Fog contributed 52.3% of leaf water for the tree community in the driest/winter month (**Figures 3A, B**). Furthermore, the contribution of soil water to leaf water for the epiphyte community was extremely limited and infrequent (<2%; **Figures 3A, B**). Thus, in summer month, leaf water in trees appears to be entirely due to soil water uptake and leaf water in epiphytes was obtained from rainfall. In the peak dry/winter season, nearly all (99.2%) the leaf water of the epiphyte community was sourced from fog, while the tree community used both soil water and fog water uptake almost equally to maintain leaf water supply (**Figure 3A**).

We computed correlations of isotope ratios of rain and fog water to soil water in the wet (July/summer) and dry (February/winter) seasons. Isotope ratios of rain and soil water showed a highly significant correlation during the wet season (R^2^ = 0.61, P<0.005; **Figures 3C–F**), when heavy rainfall occurs on a daily basis (**Figure 1**), whereas no significant correlation was observed during February (R^2^ = 0.21, p>0.05; **Figures 3C–F**), when the rainfall was nearly 40 times lower (**Figure 1**). On the other hand, isotope ratios of fog and soil water were significantly correlated in the dry/winter season (February; R^2^ = 0.45, p<0.05; **Figures 3D–F**). In comparison, there was no significant correlation between isotope ratios of fog water and soil water in the summer month (July; p>0.05; **Figures 3D–F**). In other words, rainfall is the main water input for the soil water during wet summer months, while fog contributes significantly to soil water in the dry winter season.

### 3.5 Variations in photosynthesis rate, transpiration rate, stomatal conductance, stomatal density, δ^13^C, leaf turgor loss point and leaf hydraulic capacitance for the tree and epiphyte communities in the wettest and driest months

**Figure 4:**
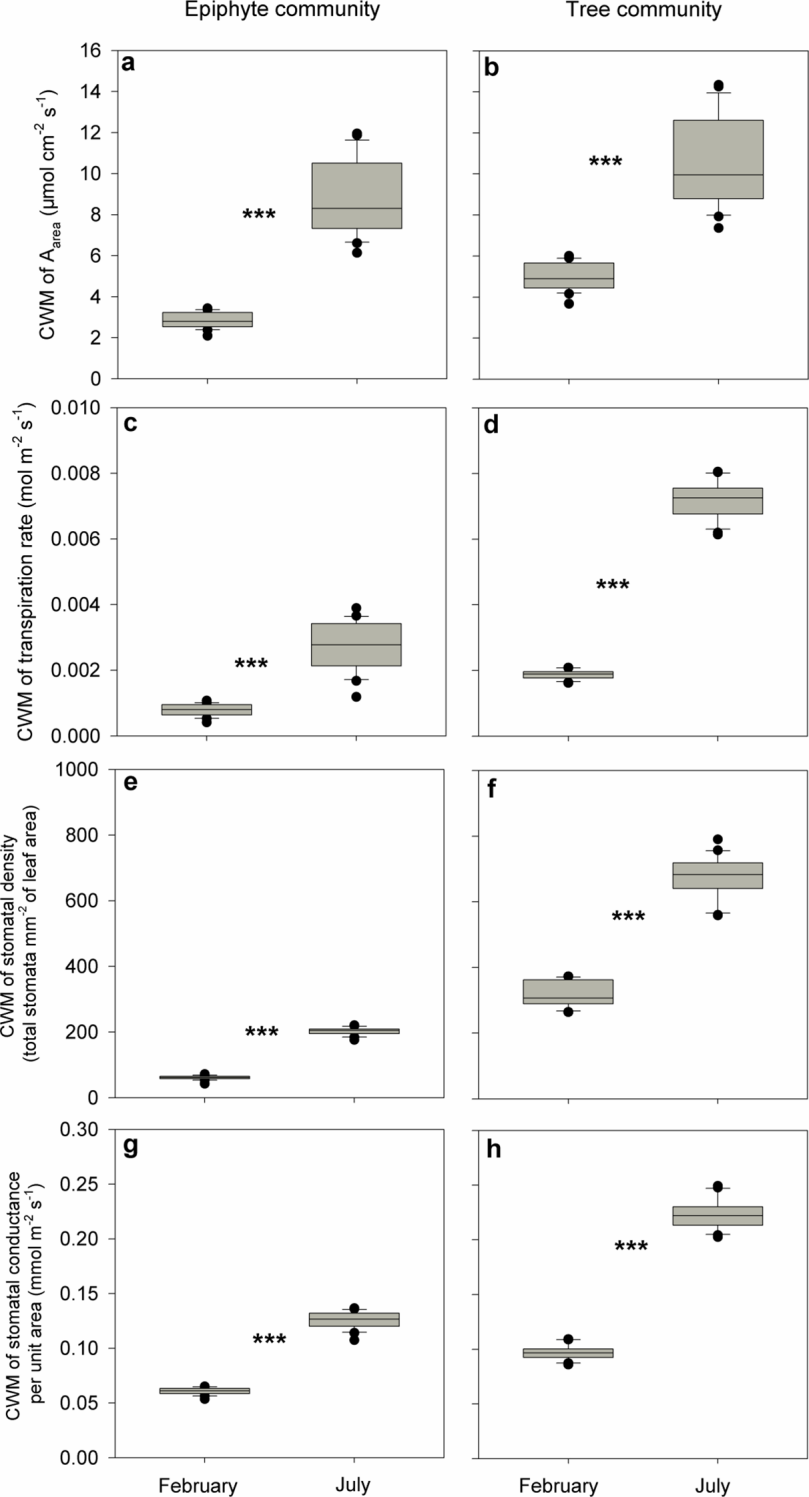
Differences in community-weighted mean values (CWM) of **(A, B)** maximum photosynthesis rate (A_area_; μmol cm^-2^ s^-1^), **(C, D)** transpiration rate (mol m^-2^ s^-1^), **(E, F)** stomatal density (total stomata mm^-2^ of leaf area), and **(G, H)** stomatal conductance (mmol m^-2^ s^-1^) and between July and February for all the tree and epiphyte species sampled. *** indicates significant differences at P<0.001 based on Wilcoxon signed-rank tests. Bars indicate the mean values and error bars denote standard errors.

We observed reduced photosynthesis and transpiration rates for both plant communities in the driest (winter) month compared to the wettest (summer) month: CWMs of photosynthesis rate in February (2.9 μmol cm^-2^ s^-1^ and 4.9 μmol cm^-2^ s^-1^ for the epiphytes and the trees, respectively) were approximately one-third to one-half of same values in July (8.2 μmol cm^-2^ s^-1^ and 10.7 μmol cm^-2^ s^-1^, for the epiphytes and the trees, respectively; p<0.001, Wilcoxon signed-rank tests, **Figures 4A, B**). Similarly, CWMs of transpiration rates for both the plant communities in February (0.001 and 0.002 mol m^-2^ s^-1^ for the epiphytes and trees, respectively) were about one-third of those in July (0.003 and 0.0067 mol m^-2^ s^-1^, respectively; p<0.001, Wilcoxon signed-rank tests, **Figures 4C, D**).

We also found a reduced stomatal density and stomatal conductance for both the plant communities in the driest (winter) month compared to the wettest (summer) month: CWMs of stomatal density for both the plant communities in February (62 stomata mm^-2^ of leaf and 316 total stomata mm^-2^ of leaf for the epiphytes and the trees, respectively) were about one-third to one-half of those in July (203 stomata mm^-2^ of leaf and 676 stomata mm^-2^ of leaf, for the epiphytes and the trees, respectively) (p<0.001, Wilcoxon signed-rank tests, **Figures 4E, F**). Similarly, CWMs of stomatal conductance in February (0.06 mmol m^-2^ s^-1^ and 0.1 mmol m^-2^ s^-1^ for the epiphytes and the trees, respectively) were approximately half of corresponding values in July (0.11 mmol m^-2^ s^-1^and 0.2 mmol m^-2^ s^-1^) for epiphytes and trees (p<0.001, Wilcoxon signed-rank tests, **Figures 4G, H**).

**Figure 5:**
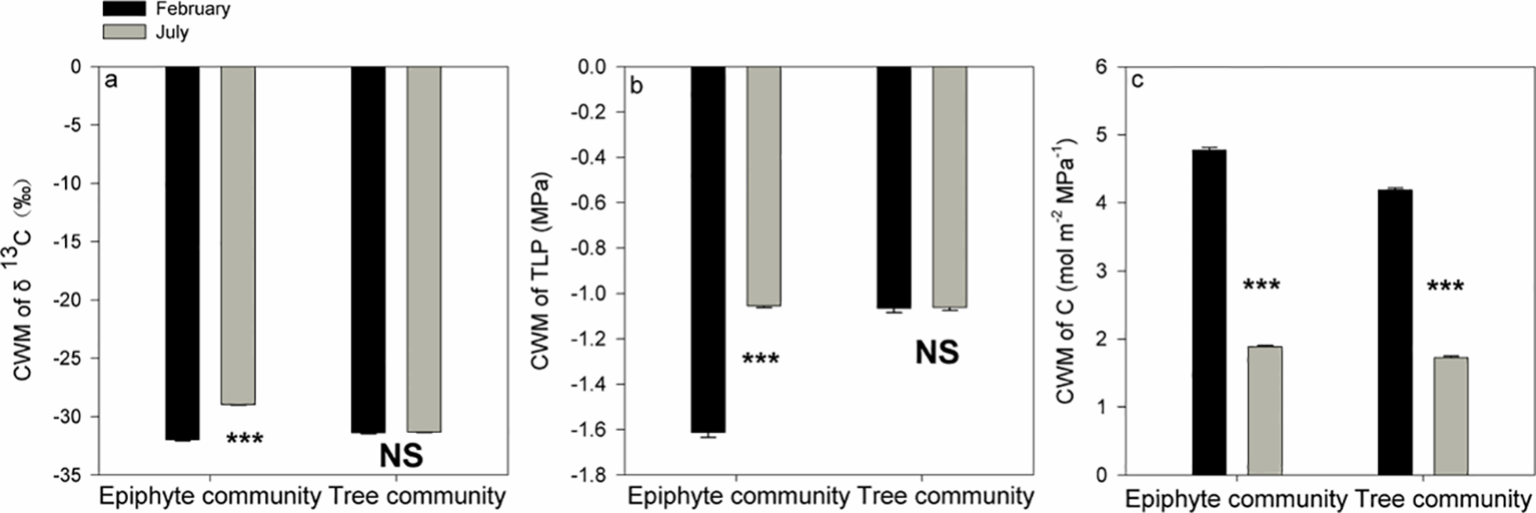
Differences in community-weighted mean values (CWM) of δ^13^C **(A)**, the leaf turgor loss point [TLP, **(B)**], and leaf hydraulic capacitance **(C)** between July and February for all the tree and epiphyte species. *** indicates significant differences at P<0.001 and NS indicates that no significant differences were noticed (P>0.05) after Wilcoxon signed-rank tests were performed. Bars indicate the mean values; error bars denote standard errors.

For the tree community, CWM values of δ^13^C were -31.32 ‰ and -31.97 ‰ for July and February respectively, and CWMs for TLP were -1.07 MPa and -1.06 MPa for July and February, respectively. The CWM values for the tree community did not differ between July and February for δ^13^C and TLP (p>0.05, Wilcoxon signed-rank tests, **Figures 5A, B**). In contrast, for the epiphyte community, the CWM values of δ^13^C (-28.95 ‰ and -31.97 ‰ in July and February, respectively) and TLP (-1.06 MPa and -1.61 MPa in July and February, respectively) were all significantly more negative (p<0.001, Wilcoxon signed-rank tests, **Figures 5A, B**), in February compared to July (**Figures 5A, B**).

A significantly increased leaf hydraulic capacitance (C) was observed for both the plant communities in the driest (winter) month as compared to the wettest (summer) month: CWMs of C in February (4.77 mol m^-2^ MPa^-1^ and 4.19 mol m^-2^ MPa^-1^ for the epiphytes and the trees, respectively) were approximately 2.2-2.5 times the corresponding values in July (1.88 mol m^-2^ MPa^-1^ and 1.72 mol m^-2^ MPa^-1^, for the epiphytes and the trees, respectively; p<0.001, Wilcoxon signed-rank tests, **Figure 5C**).

## 4 Discussion

Our study quantifies the variation in the contribution of rain, fog, and soil waters to leaf water content in an old-growth tropical cloud forest ecosystem. The data presented herein illustrate the mechanism by which this tropical cloud forest plant community sustains an adequate water supply during contrasting periods, namely, the abundant rainfall of summer versus the scarcity of precipitation during winter. We infer that when there is heavy rainfall during a summer month (wet season), large quantities of this water are added to the soil. The trees take up the water from the soil using the standard hydrological process that depends on the well-known soil-plant-atmosphere continuum (SPAC) mechanism (Berry et al., 2019; Schreel and Steppe, 2020). Along with high recharge of soil water through precipitation, other climatic conditions of high temperature, high transpiration, ample solar radiation, enriched stomatal density, stomatal conductance and enhanced photosynthesis also favor this pathway of water movement during the summer. On the other hand, in a peak (dry) winter month, we see a significant downregulation of photosynthesis and transpiration, probably caused by low temperatures and reduced solar radiation/light availability, which could reduce (transpirational) water demand, and thus soil water uptake. Changes in climatic and physiological conditions, combined with factors such as leaf wetting, are known to favor foliar water uptake (FWU; Goldsmith et al., 2013; Eller et al., 2016; Oliveira et al., 2014; Schreel and Steppe, 2020). We hypothesize that during winter days, the water requirement of tissues at the top of the canopy would be greater than that in lower parts, especially in tall trees, and this could favor FWU to maintain leaf turgor. Low transpiration losses (reduced water needs for transpiration), combined with soil water uptake and foliar fog uptake could fulfil the leaf water requirements in the dry season.

### 4.1 The relative contributions of soil water, rainfall and fog to the tree community in the wettest and the driest months

Consistent with the previous observations (Scholl et al., 2011; Brinkmann et al., 2018; Schihada et al., 2018; Zhan et al., 2020), our results clearly show that soil water, rainfall, and fog have very different isotope (δ^2^H, and δ^18^O) concentrations with the changing intensity in rainfall between seasons. Therefore, the Bayesian stable isotope mixing model could apportion the relative contributions of fog, soil water, and rainfall to leaf water for tree and epiphyte communities. We found a clear contrast between the source of leaf water in July (wet summer) and February (peak of a dry winter season), with nearly all the leaf water in trees in the wettest summer month being taken up from soils. We also found that transpiration rates were significantly high in July (>3 times that in the winter month), and the soil water being continuously replenished by the daily rainfall (indeed, the isotope signals for soil water were highly related to those for rainfall). In this scenario, it is most likely that the trees rely on the SPAC pathway (instead of foliar uptake of rainfall). On the contrary, during dry winter weather, the temperatures are low, transpiration rates are much lower, and photosynthesis is also strongly reduced. In such scenario, we infer that soil water requirement (and thus its uptake) is reduced, and a FWU (of fog) could be facilitated by trees so that the leaf turgor remains maintained.

Pathways/strategies for FWU vary across different plant species (as reviewed extensively by Berry et al., 2019, and by Schreel and Steppe, 2020), and it is plausible that the net cumulative effect of FWU at the community/ecosystem levels are far reaching than initially thought, a hypothesis that our data indicates. Further research is warranted in this direction.

By examining our results in totality, we could infer that rain is a major contributor of soil water during summers and this water is taken up by the trees by adapting a traditional SPAC flow. But during dry winters, the trees might additionally adapt a strategy involving FWU of fog to replenish leaf water, while soil water uptake may still remain the primary source of transpired water. Further, there could be potentially confounded contributions of fog to soil water to some extent. It is possible that FWU might decouple leaf-gas exchange from soil water availability (Schreel and Steppe, 2020), thus further complicating the quantification of the different sources to leaf water. It is also conceivable that trees might also tap deep underground water in the driest month, but some scholars have already reported that this tropical cloud forest has very shallow soil (less than 30 cm) and short root length (less than 30 cm) (Long et al., 2011c; Yang et al., 2021), therefore, this is very unlikely (Hildebrandt and Eltahir, 2007).

Surprisingly, we found that TLP and δ^13^C do not differ between the peak wet and dry seasons even though TLP and δ^13^C are good indicators of general water availability or water stress (Farrell et al., 2017; Acosta-Rangel et al., 2018). Also, these are highly associated with photosynthesis rates, as they influence the ability of plants to maintain cell turgor under drought conditions and reflect long-term water-use efficiency (Nogueira et al., 2004; Bartlett et al., 2012). The stable TLP and δ^13^C values for the tree community and comparable soil water content between July and February indicate adequate water availability to the trees in both seasons. Any difference in water availability between seasons may in part be compensated by differences in leaf hydraulic capacitance (Goldsmith et al., 2013). The observation that leaf hydraulic capacitance in the dry season was 2.5 times than in the wet season indicates modification of leaf traits for better water retention in the dry season. All things considered, the low rainfall input in the driest month could have been compensated with increased leaf hydraulic capacitance, suppressed photosynthesis rate and transpiration, low temperature, high humidity, and possibly a fog input. These conditions appear sufficient to maintain adequate water availability for this cloud forest, at least for the tree and epiphyte communities (discussed below).

There remain some gaps in our understanding of water cycling for the tree community. We need further investigation to understand the mechanism of FWU for tree community in the dry season. We also need specific data on leaf temperature, leaf water potential, soil water potential, and sap flux measurements to understand the direction and flow rates of water movement. The effects of leaf phenology should also be considered in interpreting the effects of variation in solar radiation, temperature, and rainfall on this tropical cloud forest ecosystem. To assess fog inputs, we need data on fog intensity and duration, above-canopy observations of climatic variables, wind-driven rain and continuous soil water content measurements alongside sap-flow observations of transpiration (Holwerda et al., 2006; Schmid et al., 2011).

### 4.2 The relative contributions of rainfall, fog, and soil water to epiphyte community across the wettest and driest months

Given that the epiphyte community on Hainan Island derives its water fully relies on atmospheric sources, such as fog and rainfall, which could be directly absorbed by their leaves and roots, and could serve as leaf water resources in both the seasons (Gotsch et al., 2015, 2018; Wu et al., 2018). Although, fog and rainfall are equally frequent in July, rainfall can provide much higher water input than fog in the wet season. Moreover, photosynthesis rate and transpiration for epiphyte community are relatively high in July, which should favor root water absorption. In a peak dry winter period, fog still occurs every day, and the resultant leaf wetting should favor its uptake.

In February the epiphyte community has lower TLP than that in July. Further, the low temperature, and limited and infrequent rainfall in the driest month may cause epiphytes to lower their osmotic potential and to increase the uptake of fog water (Bartlett et al., 2014; Gotsch et al., 2015, 2018). Indeed, we observed that fog acted as a main source for leaf water for the epiphyte community in the driest month. We also found δ^13^C in February was higher than that in July, indicating higher water use efficiency under lower water availability (Acosta-Rangel et al., 2018). However, water use efficiency could also be affected by the changes in the photosynthesis rates (Nogueira et al., 2004), and the suppressed photosynthetic rates in February could have counteracted the increase in water use efficiency to a certain extent.

### 4.3 Differences in community-level hydraulic responses between seasons and life-forms

Previous studies have documented reduced photosynthesis and transpiration rates during the arid winter season, but limited to a select few species (Burgess and Dawson, 2004; Goldsmith et al., 2013; Alvarado-Barrientos et al., 2014). We show that community level ecophysiological responses (such as reduced photosynthesis rate and transpiration, and fog utilization) vary with the quantity of rainfall and life-forms (trees *vs.* epiphytes). These findings therefore expand prior knowledge on the varied hydraulic responses as a function of water availability and life-forms from the species level (Oliveira et al., 2014; Gotsch et al., 2015; Wu et al., 2018; Berry et al., 2019) to the community level.

We also found that fog had different effects on tree and epiphyte communities. Given that the epiphytes here cannot tap into the host tree xylem water, foliar uptake of fog water could be critical to ensure leaf water supply for the epiphyte community when rainfall is critically low in the driest month. However, reduction in temperature and suppression of photosynthesis might result in lower leaf water use efficiency and higher leaf turgor loss point for the epiphyte community. Fog contributed to leaf water supply for tree community in the cold dry month, when rainfall was limited, and was therefore important for the tree community as well.

Detailed knowledge of the hydrological feedbacks of tropical forest ecosystems is only emerging (Staal et al., 2020), but there are predictions of dramatic losses (>50%) of tropical cloud forest cover due to climate warming and increase in cloud-base heights [predictions for 2052 by IPCC 5th assessment reports; as well as studies by (Los et al., 2019)]. Another independent simulation study predicts significant loss of tropical cloud forests in Mexico by 2080 if temperatures were to rise by >4°C and surrounding lowland forests were lost (Ponce-Reyes et al., 2012). We found that fog water made little contribution to tree and epiphyte communities in the wettest month, reasons of which need further investigations, which are out of scope of this report. However, in the peak of the dry season, fog appears to be important for water safety, hence the importance of maintaining cloud immersion for cloud forest persistence (Wu et al., 2018).

Forest ecosystems provide the most sustainable and highest quality freshwater (Vose, 2019) and soil water content appears to be the key determinant of freshwater supply from forest ecosystems (Neary et al., 2009). Sustaining this important ecohydrological process involves maintaining the structural and functional integrity of the cloud forest ecosystem and avoiding serious climatic changes (Zhan et al., 2020).

## 5 Conclusions

We conclude that both rainfall and aerial water such as fog, are important components of water budget safety for tree and epiphyte species in the cloud forests of Hainan Island. Further, reductions in solar radiation and atmospheric temperature, and a strong dry season results in two key ecosystem hydraulic responses: (i) reduced photosynthesis and transpiration, which might induce a large reduction in soil water uptake, and (ii) enhanced leaf hydraulic capacitance and foliar water uptake. These are the crucial factors that help tropical cloud forest plant species to operate within the hydraulic safety margins and maintain adequate water under seasonal changes in water supply. This ecohydrological relationship should be preserved in order to ensure freshwater supply across the wettest and driest months. The sensitivity of cloud-base heights to climate changes puts the cloud forest at great risk (Bruijnzeel et al., 2011; Sarmiento and Kooperman, 2019). Precise data on ecophysiological responses of tree and epiphyte species to changing climatic conditions are urgently needed to be incorporated in climate-vegetation models for tropical cloud forest.

## Data Availability

The original contributions presented in the study are included in the article/**Supplementary Material**. Further inquiries can be directed to the corresponding authors.
