# Supplementary material for: The contributions of rainfall and fog to leaf water of tree and epiphyte communities in a tropical cloud forest
Source: Front Plant Sci. 2024 Oct 17;15:1488163. doi: 10.3389/fpls.2024.1488163 (PMC11524870; doi:10.3389/fpls.2024.1488163)
Supplement: Supplementary file 1 [file DataSheet1.doc]

**Supplementary Data Sheet - Text S1. Method for measuring epiphyte species abundance**

Host trees with a diameter at breast height (DBH) greater than 20 cm that had crowns that were not easily visible from the ground were climbed using single-rope climbing techniques (where deemed safe). Host trees with DBH smaller than 20 cm and height higher than 3m were surveyed with binoculars and a thief rod (Perry, 1978), while small trees (height < 3 m) were surveyed from the ground. Samples of unknown vascular epiphyte species were collected and brought back to lab for identification according using the Flora Republicae Popularis Sinicae (Editorial Committee of Flora of China, 2015). In this study, clusters of vascular epiphytic plants were identified according to the approach by Sanford, (1968): if the intermingling individual stems belonging to conspecific plants were spatially separated and distinguishable from each other, these were classified as different individuals, while a collection of individuals consisting of more than one species, each was classified as different species.

**Supplementary Data Sheet - Text S2. Corrections for xylem water of *δ*D and *δ*^18^O**

The correction procedures for leaf water of δ^2^H and *δ*^18^O were given by Schultz *et al*. (2011). The correct curves were created by Wu *et al.* (2016), which were:

δ^2^H=0.25(*ln NB*)^2^-0.46 (*ln NB*)+1.18 (R^2^ = 0.993, *p*<0.001)

δ^18^O=0.17 (*ln NB*)^2^-0.29 (*ln NB*)+0.73 (R^2^ = 0.994, *p*<0.001)

δ^18^O=-6.33*BB*+6.38 (R^2^ = 0.884, *p*<0.001)

**where NB (narrow band absorber metric) represents a correction for methanol contamination, and BB (broad band absorber metric) indicates a correction for ethanol contamination.** Furthermore, to test the accuracy of the corrections for leaf water, isotopic data (δ^2^H and δ^18^O) from Isotopic Ratio Infrared Spectroscopy (IRIS) analysis were compared with those from Isotope Ratio Mass Spectrometry (IRMS) (Finnigan MAT253, Thermo Finnigan, Bremen,Germany, Stable Isotope Ratio Mass Spectrometer Laboratory of Chinese Academy of Forestry).

**References**

Editorial Committee of Flora of China., 2015. Flora Reipublicae Popularis Sinicae (Chinese edition of Flora of China). Beijing: Science Press. Available: http://www.efloras.org.

Perry, G.R., 1978. A method of access into the crowns of emergent and canopy trees. Biotropica. 10, 155-157. https:// 10.2307/2388019.

Sanford, W.W., 1968. Distribution of epiphytic orchids in semideciduous tropical forest in southern Nigeria. J. Ecol. 56, 697-705. https:// 10.2307/2258101.

Schultz, N.M., Griffis, T.J., Lee, X., Baker, J.M., 2011. Identification and correction of spectral contamination in 2H/1H and 18O/16O measured in leaf, stem, and soil water. Rapid. Commun. Mass. Spectrom. 25, 3360-3368. https:// 10.1002/rcm.5236.

Wu, Y., Liang, S., Liu, W., Liu, W., Su, L., Fu, P., Lu, H., 2018. Fog Water Is Important in Maintaining the Water Budgets of Vascular Epiphytes in an Asian Tropical Karst Forests during the Dry Season. Forests.9, 260-267.

https://10.3390/f9050260.

**Table S1.** Elevations ( m a.s.l.) for each of the 21 plots in our tropical cloud forest.

|  | Elevation |
| --- | --- |
| Plot 1 | 1395.24 |
| Plot 2 | 1390.26 |
| Plot 3 | 1365.22 |
| Plot 4 | 1353.24 |
| Plot 5 | 1349.18 |
| Plot 6 | 1341.21 |
| Plot 7 | 1337.23 |
| Plot 8 | 1335.25 |
| Plot 9 | 1328.26 |
| Plot 10 | 1325.24 |
| Plot 11 | 1332.21 |
| Plot 12 | 1326.22 |
| Plot 13 | 1329.24 |
| Plot 14 | 1326.24 |
| Plot 15 | 1318.25 |
| Plot 16 | 1325.26 |
| Plot 17 | 1317.24 |
| Plot 18 | 1319.27 |
| Plot 19 | 1320.26 |
| Plot 20 | 1316.25 |
| Plot 21 | 1313.24 |

**Table S2.** Relative abundances (%) for each of the 60 tree species and 30 epiphyte species found in 21 400 m^2^ plots.

| **Functional group** | **Species name** | **Family name** | **Relative abundance (%)** |
| --- | --- | --- | --- |
| Tree species | *Distylium racemosum* | *Hamamelidaceae* | 11.96 |
| Tree species | *Psychotria rubra* | *Rubiaceae* | 9.69 |
| Tree species | *Syzygium buxifolium* | *Myrtaceae* | 6.98 |
| Tree species | *Ervatamia officinalis* | *Apocynaceae* | 5.51 |
| Tree species | *Symplocos poilanei* | *Symplocaceae* | 5.35 |
| Tree species | *Engelhardtia roxburghiana* | *Juglandaceae* | 4.63 |
| Tree species | *Symplocos lancifolia* | *Symplocaceae* | 4.60 |
| Tree species | *Osmanthus didymopetalus* | *Oleaceae* | 4.44 |
| Tree species | *Melastoma penicillatum* | *Melastomataceae* | 4.21 |
| Tree species | *Rhododendron moulmainense* | *Ericaceae* | 3.49 |
| Tree species | *Gordonia axillaris* | *Theaceae* | 3.33 |
| Tree species | *Michelia mediocris* | *Magnoliaceae* | 2.20 |
| Tree species | *Illicium ternstroemioides* | *Schisandraceae* | 1.97 |
| Tree species | *Pentaphylax euryoides* | *Pentaphylacaceae* | 1.89 |
| Tree species | *Elaeocarpus howii* | *Elaeocarpaceae* | 1.88 |
| Tree species | *Schefflera octophylla* | *Araliaceae* | 1.81 |
| Tree species | *Cinnamomum tsoi* | *Lauraceae* | 1.66 |
| Tree species | *Cyclobalanopsis disciformis* | *Fagaceae* | 1.62 |
| Tree species | *Polyalthia plagioneura* | *Annonaceae* | 1.39 |
| Tree species | *Osmanthus hainanensis* | *Oleaceae* | 1.26 |
| Tree species | *Lithocarpus hancei* | *Fagaceae* | 1.24 |
| Tree species | *Diplospora dubia* | *Rubiaceae* | 1.22 |
| Tree species | *Machilus velutina* | *Lauraceae* | 1.14 |
| Tree species | *Syzygium araiocladum* | *Myrtaceae* | 1.10 |
| Tree species | *Viburnum hainanense* | *Adoxaceae* | 1.09 |
| Tree species | *Rapanea neriifolia* | *Primulaceae* | 1.05 |
| Tree species | *Neolitsea pulchella* | *Lauraceae* | 1.02 |
| Tree species | *Syzygium odoratum* | *Myrtaceae* | 0.96 |
| Tree species | *Dendropanax dentiger* | *Araliaceae* | 0.92 |
| Tree species | *Cryptocarya chinensis* | *Lauraceae* | 0.82 |
| Tree species | *Podocarpus neriifolius* | *Podocarpaceae* | 0.77 |
| Tree species | *Cyclobalanopsis fleuryi* | *Fagaceae* | 0.76 |
| Tree species | *Acronychia oligophlebia* | *Rutaceae* | 0.69 |
| Tree species | *Cleyera incornuta* | *Pentaphylacaceae* | 0.68 |
| Tree species | *Cyclobalanopsis poilanei* | *Fagaceae* | 0.67 |
| Tree species | *Dacrydium pierrei* | *Podocarpaceae* | 0.62 |
| Tree species | *Schima superba* | *Theaceae* | 0.55 |
| Tree species | *Ardisia quinquegona* | *Primulaceae* | 0.51 |
| Tree species | *Neolitsea cambodiana* | *Lauraceae* | 0.44 |
| Tree species | *Ilex lancilimba* | *Aquifoliaceae* | 0.43 |
| Tree species | *Ficus variolosa* | *Moraceae* | 0.40 |
| Tree species | *Microtropis submembranacea* | *Celastraceae* | 0.40 |
| Tree species | *Symplocos sumuntia* | *Symplocaceae* | 0.38 |
| Tree species | *Lithocarpus silvicolarum* | *Fagaceae* | 0.31 |
| Tree species | *Machilus suaveolens* | *Lauraceae* | 0.29 |
| Tree species | *Pittosporum balansae* | *Pittosporaceae* | 0.29 |
| Tree species | *Exbucklandia tonkinensis* | *Hamamelidaceae* | 0.24 |
| Tree species | *Ilex hainanensis* | *Aquifoliaceae* | 0.21 |
| Tree species | *Myrica rubra* | *Myricaceae* | 0.18 |
| Tree species | *Xanthophyllum hainanense* | *Polygalaceae* | 0.16 |
| Tree species | *Elaeocarpus sylvestris* | *Elaeocarpaceae* | 0.13 |
| Tree species | *Lithocarpus chiungchungensis* | *Fagaceae* | 0.08 |
| Tree species | *Casearia membranacea* | *Salicaceae* | 0.07 |
| Tree species | *Ilex kobuskiana* | *Aquifoliaceae* | 0.07 |
| Tree species | *Gomphandra tetrandra* | *Stemonuraceae* | 0.06 |
| Tree species | *Symplocos ovatilobata* | *Symplocaceae* | 0.04 |
| Tree species | *Lithocarpus fenzelianus* | *Fagaceae* | 0.04 |
| Tree species | *Aidia canthioides* | *Rubiaceae* | 0.03 |
| Tree species | *Podocarpus imbricatus* | *Podocarpaceae* | 0.02 |
| Tree species | *Parakmeria lotungensis* | *Magnoliaceae* | 0.02 |
| Epiphyte species | *Bulbophyllum retusiusculum* | *Orchidaceae* | 31.31 |
| Epiphyte species | *Liparis delicatula* | *Orchidaceae* | 22.16 |
| Epiphyte species | *Coelogyne fimbriata* | *Orchidaceae* | 18.52 |
| Epiphyte species | *Eria thao* | *Orchidaceae* | 6.30 |
| Epiphyte species | *Elaphoglossum conforme* | *Dryopteridaceae* | 3.64 |
| Epiphyte species | *Pholidota chinensis* | *Orchidaceae* | 3.64 |
| Epiphyte species | *Pyrrosia eberhardtii* | *Polypodiaceae* | 3.40 |
| Epiphyte species | Psychotria serpens | *Rubiaceae* | 1.97 |
| Epiphyte species | *Humata repens* | *Davalliaceae* | 1.89 |
| Epiphyte species | *Pholidota yunnanensis* | *Orchidaceae* | 1.61 |
| Epiphyte species | *Lepidogrammitis rostrata* | *Polypodiaceae* | 1.19 |
| Epiphyte species | *Dendrobium sinense* | *Orchidaceae* | 0.75 |
| Epiphyte species | *Epigeneium clemensiae* | *Orchidaceae* | 0.52 |
| Epiphyte species | *Bulbophyllum ledungense* | *Orchidaceae* | 0.51 |
| Epiphyte species | *Neottopteris nidus* | *Aspleniaceae* | 0.47 |
| Epiphyte species | *Eria obvia* | *Orchidaceae* | 0.41 |
| Epiphyte species | *Bulbophyllum ambrosia* | *Orchidaceae* | 0.29 |
| Epiphyte species | *Dendrobium densiflorum* | *Orchidaceae* | 0.23 |
| Epiphyte species | *Eria quinquelamellosa* | *Orchidaceae* | 0.23 |
| Epiphyte species | *Vittaria flexuosa* | *Pteridaceae* | 0.17 |
| Epiphyte species | *Pseudodrynaria coronans* | *Drynariaceae* | 0.16 |
| Epiphyte species | *Schoenorchis gemmata* | *Orchidaceae* | 0.15 |
| Epiphyte species | *Dendrobium williamsonii* | *Orchidaceae* | 0.14 |
| Epiphyte species | *Davallia mariesii* | *Davalliaceae* | 0.13 |
| Epiphyte species | *Bulbophyllum kwangtungense* | *Orchidaceae* | 0.09 |
| Epiphyte species | *Selaginella tamariscina* | *Selaginellaceae* | 0.03 |
| Epiphyte species | *Hoya lancilimba* | *Apocynaceae* | 0.03 |
| Epiphyte species | *Lepisorus thunbergianus* | *Polypodiaceae* | 0.02 |
| Epiphyte species | *Phlegmariurus phlegmaria* | *Huperziaceae* | 0.02 |
| Epiphyte species | *Cleisostoma paniculatum* | *Orchidaceae* | 0.01 |

**Table S3.** Wilcoxon rank sum test results for the differences in δ^2^H and δ^18^O among soil, precipitation, tree community and epiphyte community in both the wet and dry seasons. Bold text indicates P<0.001 based on Wilcoxon signed-rank tests. W and P indicates test statistic and p-value, respectively for Wilcoxon rank sum test

|  | Wet season | | | | Dry season | | | |
| --- | --- | --- | --- | --- | --- | --- | --- | --- |
|  | δ^2^H | | δ^18^O | | δ^2^H | | δ^18^O | |
|  | W | P | W | P | W | P | W | P |
| **Soil water *vs.* rainfall** | **0** | **<0.001** | **0** | **<0.001** | **0** | **<0.001** | **68** | **<0.001** |
| **Soil water *vs.* fog water** | **0** | **<0.001** | **0** | **<0.001** | **61** | **<0.001** | **110** | **<0.001** |
| **Soil water *vs.* tree community** | 220.5 | >0.05 | 220.5 | >0.05 | **141.5** | **<0.001** | **0** | **<0.001** |
| **Soil water *vs.* epiphyte community** | **0** | **<0.001** | **0** | **<0.001** | **131.5** | **<0.001** | **0** | **<0.001** |
| **Rainfall *vs.* fog water** | **441** | **<0.001** | **441** | **<0.001** | **0** | **<0.001** | **46** | **<0.001** |
| **Rainfall *vs*. tree community** | **441** | **<0.001** | **441** | **<0.001** | **0** | **<0.001** | **0** | **<0.001** |
| **Rainfall *vs.* epiphyte community** | 292 | >0.05 | 255 | >0.05 | **0** | **<0.001** | **0** | **<0.001** |
| **Fog water *vs.* tree community** | **441** | **<0.001** | **441** | **<0.001** | **52** | **<0.001** | **0** | **<0.001** |
| **Fog water *vs.* epiphyte community** | **0** | **<0.001** | **81** | **<0.001** | 190 | >0.05 | 175 | >0.05 |
| **tree community *vs.* epiphyte community** | **0** | **<0.001** | **0** | **<0.001** | **41** | **<0.001** | **0** | **<0.001** |

**Fig. S1.** The monthly variations in mean temperature (℃), precipitation (mm) and wind speed (m s^-1^) in year 2018.





**Fig. S2.** The location of automatic weather station and the 21 plots in the tropical forest.


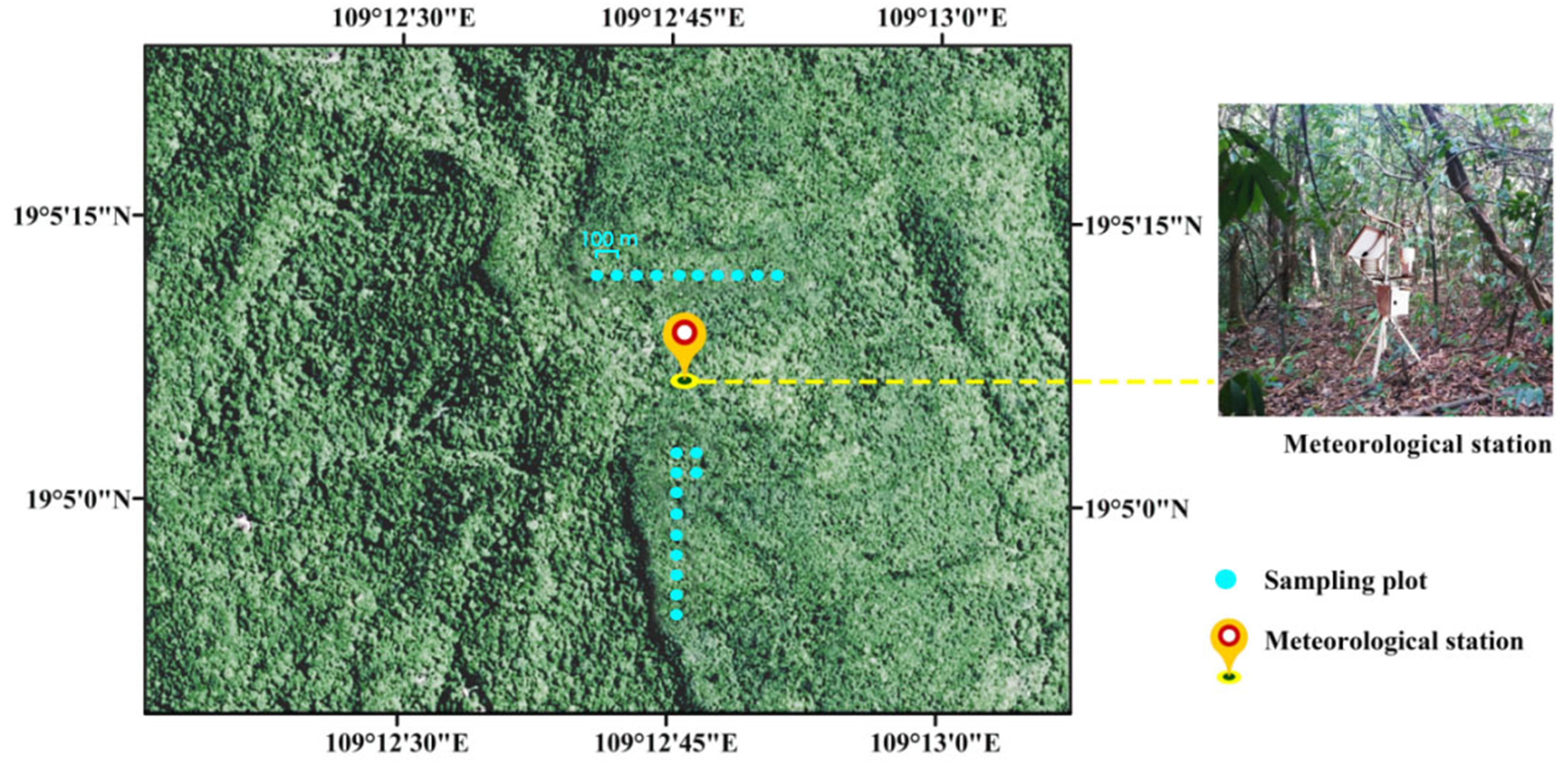


**Fig. S3.** The picture of our self-made simple fog drip collector (c) whose principle is the same as the commonly used fog drip collector (a). Fog drip can be deposited in the bottom of the plastic sheet which is hanged between two adjacent trees (b and c).

**
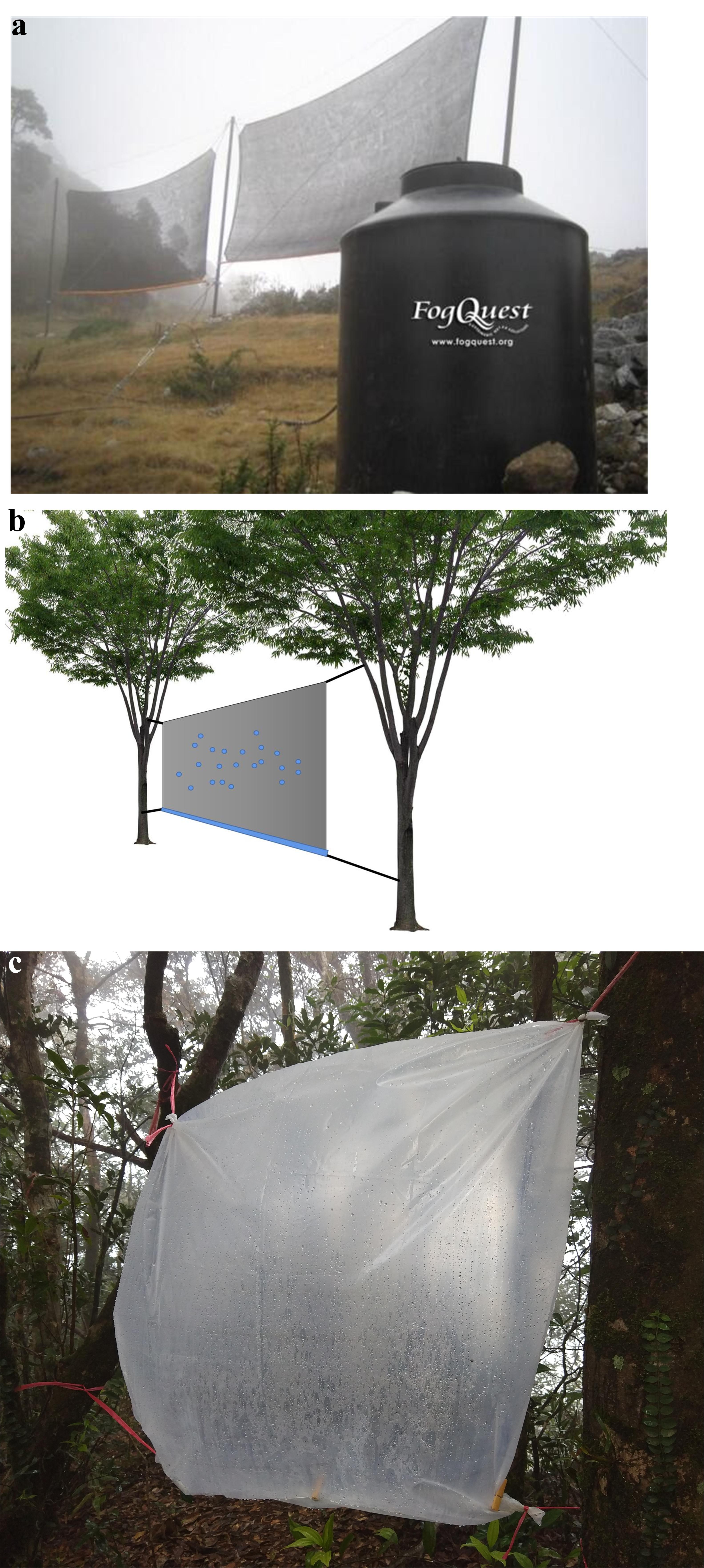
**
